# E3 ubiquitin ligase NKLAM/RNF19b suppresses myc-driven B cell lymphomagenesis in Eμ-myc mice

**DOI:** 10.3389/fonc.2026.1874366

**Published:** 2026-07-15

**Authors:** Richard G. Hoover, Emily C. Matchett, Jacki Kornbluth

**Affiliations:** 1 Department of Pathology, Saint Louis University School of Medicine, St. Louis, MO, United States; 2 Research and Development Service, St. Louis VA Medical Center, St. Louis, MO, United States

**Keywords:** B cell lymphoma, immunoediting, lymphomagenesis, MYC, NKLAM, ubiquitin ligase

## Abstract

**Introduction:**

Natural Killer Lytic-Associated Molecule (NKLAM) (also known as RNF19b) is a membrane-bound E3 ubiquitin ligase. Previous studies demonstrated a role of NKLAM in natural killer (NK) cell function and pro-inflammatory cytokine production by macrophages. Studies using lymphoma, melanoma and breast cancer cells found that tumor dissemination and metastasis are greater in NKLAM-deficient knockout (KO) mice than in wild type (WT) mice, indicating that NKLAM participates in controlling tumor development *in vivo*.

**Methods:**

We employed the Eμ-myc mouse model to determine whether NKLAM influences B cell lymphomagenesis. In young Eμ-myc mice, overexpression of myc in B lineage cells leads to expansion of non-neoplastic precursor B cells, which subsequently disappear. Over time, pre-B, pro-B or immature B cell lymphomas develop.

**Results:**

NKLAM KO Eμ-myc mice have higher levels of precursor B cells than WT transgenic mice. These cells express more myc and Bcl-2 and persist longer. Lymphomas develop more rapidly in NKLAM KO Eμ-myc mice and strikingly, have a more differentiated phenotype, characterized by surface IgM. These lymphomas are also less aggressive than IgM- tumors when injected into non-transgenic mice. Infusion of NKLAM+ immune cells into young NKLAM KO Eμ-myc mice extends their survival, which increases the proportion of mice that develop more aggressive, immune-resistant IgM- lymphomas.

**Discussion:**

These studies indicate that NKLAM contributes to both the early and late phases of myc-driven B cell lymphomagenesis, first by limiting expression of myc and Bcl-2 within the non-malignant pre-B cells, resulting in longer tumor-free survival. This is followed by immunoediting by NKLAM+ immune cells, leading to development of IgM- lymphomas that are less immunogenic and more aggressive.

## Highlights

- NKLAM deficiency accelerates myc-driven B cell lymphomagenesis.
- NKLAM limits expression of myc and Bcl-2 in non-neoplastic B cell progenitors in Eµ-myc mice.
- Infusion of NKLAM+ immune cells into young NKLAM-deficient Eµ-myc mice extends their survival, allowing for the development of less immunogenic and more aggressive B cell lymphomas.

## Introduction

Natural killer (NK) cells kill tumor cells, primarily through release of cytotoxic granules containing perforin and granzymes. They are also potent producers of interferon (IFN)-γ and tumor necrosis factor (TNF)-α which contribute to inflammatory and immune responses (1–3). NK-mediated killing is augmented by cytokines, including interleukin (IL)-2, IL-12, IL-15 and IFN (4, 5). The expression of Natural Killer Lytic-Associated Molecule (NKLAM), also known as RNF19b, is highly correlated with NK cytolytic activity (6, 7). Upon NK activation, NKLAM is rapidly transcribed, translated and embedded into cytotoxic granule membranes.

NKLAM is also upregulated in macrophages exposed to Gram positive and negative bacteria, bacterial products like lipopolysaccharide and lipotechoic acid, and interferons α, β and γ. This leads to enhanced macrophage-mediated bacterial degradation (8–10). Gene Expression Omnibus microarray analyses show elevation of NKLAM mRNA levels in monocytes exposed to several microorganisms, including *Borrelia burgdorferi*, *Chlamydia pneumoniae*, *Francisella tularensis*, *M. tuberculosis* and upon infection with several adenoviruses and rhinoviruses that cause respiratory infections.

NKLAM is a RING1-In Between Ring-RING2 (RBR) E3 ubiquitin ligase (11–13). Ubiquitination is a post-translational protein modification responsible for proteasomal degradation, lysosomal targeting, kinase and transcription factor activation, and DNA repair (14). There are hundreds of E3 ubiquitin ligases, but only 14 RBR ligases, and these are highly conserved among species (15–19).

We generated NKLAM-deficient (knockout; KO) mice to evaluate the role of NKLAM *in vitro* and *in vivo*. NKLAM KO NK cells are defective in lysing tumor cells both *in vitro* and *in vivo* (6, 20, 21). NKLAM KO mice have more pulmonary nodules than wild type (WT) mice after injection with B16 melanoma cells (20). Primary tumor growth, dissemination and metastasis of RMA-S lymphoma cells and E0771 breast cancer cells are accelerated and greater in NKLAM KO mice than in WT mice. Therefore, NKLAM plays a significant role in controlling tumor development *in vivo* (21).

NKLAM KO mice have a diminished response to both *Streptococcus pneumoniae* and Sendai virus infections. Both pneumonia models demonstrate that the lack of NKLAM results in significantly lower pro-inflammatory cytokine and chemokine levels in the lungs and plasma of infected mice (9, 10).

We employed the Eμ−myc transgenic mouse model to determine whether NKLAM influences early events of tumorigenesis. Constitutive myc expression in the B cell lineage results in development of B cell lymphomas in 100% of these mice (22–24). Young Eμ-myc mice have a polyclonal expansion of non-malignant precursor B cells, which disappear with age. B cell lymphomas emerge by 4–6 months of age. NKLAM KO and WT Eμ-myc mice were generated and studied in parallel. Tumor development is significantly accelerated in NKLAM KO Eμ-myc mice compared to WT Eμ-myc mice. There is a more robust and extended pre-B cell expansion phase and a shift in the phenotype of lymphomas that develop in NKLAM KO Eμ-myc mice compared to WT transgenic mice. These results indicate unique roles for NKLAM during both early and late stages of myc-driven B cell lymphomagenesis.

## Materials and methods

### Mice

Mice were maintained in pathogen free barrier housing and experiments were conducted in accordance with both SLU and VA institutional animal care and use guidelines, with approval from both SLU and VA Institutional Care and Use Committees (IACUC). NKLAM KO and WT C57BL/6 (B6) mice were bred in house. Male Eμ-myc mice were purchased from Jackson Laboratory (JAX) (B6.Cg-Tg(IghMyc)22Bri/J; Strain #002728). Female WT or NKLAM KO B6 mice were crossed with male Eμ-myc mice; progeny were genotyped for the myc transgene by PCR using tail DNA and primers as described by JAX. WT and NKLAM KO myc transgenic mice were bred in house in parallel, to maintain age-matched cohorts. `Blood (≤ 25 μl) was collected in EDTA tubes by submandibular bleeding. Both male and female mice were included in this study. Euthanasia was performed by exposure to CO_2_ gas at a flow rate of 3-4L/minute, followed by cervical dislocation, following IACUC guidelines.

### Isolation of pre-B and B cells

CD19+ pre-B and B cells were isolated to >95% purity from the blood of NKLAM KO and WT non-transgenic and Eμ-myc mice using Miltenyi Biotec anti-CD19 antibody-coupled magnetic beads (#130-052-201) and MACs columns (#130-042-401).

### Isolation of splenic lymphocytes

Spleens were harvested, gently homogenized and layered over a Lympholyte-M density gradient (Cedarlane, Accurate Chemical Corporation #CL5031) to remove red blood cells, dead cells and debris. Lymphocytes at the interface were collected, washed twice, resuspended in PBS, and counted.

NK cells were isolated from splenic lymphocytes using Miltenyi Biotec anti-CD49b (DX5)-coupled magnetic beads (#130-052-501) and MACs columns. Flow-through cells constituted the NK-depleted (NK-) population.

### Flow cytometry

Two x 10^5^–2 x 10^6^ blood or lymphoma cells in 0.05 ml of flow buffer (PBS + 1% FBS + 0.1% sodium azide) were incubated with 1 μl of Fc block (BD Pharmingen #553142 RRID: AB_394657) for 10 min at 4°C. Fluorescent conjugated cell surface-specific antibodies were added and incubated for an additional 30 min at 4°C. Aliquots of cells were resuspended in annexin binding buffer and stained with PE annexin V and 7-AAD. BD FACS™ Lysing Solution (BD Biosciences (#349202) eliminated red cells from blood samples. Fluorescent Accucheck counting beads (Invitrogen #10110533) were added for quantitation of cell numbers or cells were prepared for Ki-67 intracellular staining using the BD Cytofix/Cytoperm Plus Fixation/Permeabilization Kit (#55508). After staining, lymphoma cells were washed with flow buffer and fixed with 0.2 ml PBS + 1% formaldehyde. Cells were analyzed on a Becton Dickinson FACScaliber flow cytometer. BD Antibodies: PerCP anti-CD45 (#557235 RRID: AB_396609), APC anti-CD19 (#550992 RRID: AB_398483), A647 anti-Ki-67 (#561126 RRID: AB_10611874), PE anti-IgM (#553521 RRID: AB_394902), FITC anti-IgM (#553520 RRID: AB_394901), BV421 anti-CD43 (#562958 RRID: AB_2665409), PE anti-DX5 (#553858 RRID: AB_395094), APC anti-NK1.1 (#550627 RRID: AB_398463), PECy7 anti-NK1.1 (#552878 RRID: AB_394507), FITC anti-CD3 (#553061 RRID: AB_394594), A700 anti-CD3 (#557984 RRID: AB_396972), PE anti-CD314 (NKG2D) (#558403 RRID: AB_647201), PE Cy7 anti-B220 (#552772 RRID: AB_394458). BioLegend Antibodies: PEDazzle 594 anti-CD127 (#135031 RRID: AB_2564216), A488 anti-DX5 (#108913 RRID: AB_492879). eBioscience Antibodies: eFluor710 anti-MHC class I (#46-5958–82 RRID: AB_2016714), eFluor450 anti-CD3 (#48-0032–82 RRID: AB_1272193). PE Annexin V was from BioLegend (#640908) and 7-AAD was from BD (#559925 RRID: AB_2869266). NKG2D ligands (NKG2DL) were identified using mouse NKG2D/CD314 Fc chimera (R&D Systems # MAB139, RRID: AB_2133263) and PE-conjugated human IgG Fc (R&D Systems #FAB110P RRID: AB_3645670). Samples were analyzed with FlowJo software (TreeStar RRID: SCR_008520).

### Tumor injections

Mice were injected intravenously (iv) with 2 x 10^5^ lymphoma cells from NKLAM KO or WT Eμ-myc mice and monitored for tumor development daily.

### Adoptive transfer experiments

Splenic lymphocytes from non-transgenic WT or NKLAM KO mice were resuspended in PBS, and 4–5 x 10^7^ cells were injected iv into mice. NKLAM KO Eμ-myc mice were injected iv with either 5 x 10^6^ column-purified WT DX5+ NK cells or the column flow-through (NK-) lymphocytes. Injections were given every other week, starting 4 weeks after birth.

### Injection of splenic lymphocytes into livers

2.5 x 10^7^ WT splenic lymphocytes were injected into the livers of 10-11-day old NKLAM KO Eμ-myc mice, followed by a second iv injection 2 weeks later. Mice were monitored for tumor development daily.

### PCR

RNA was extracted using the RNeasy Mini Kit (Qiagen #74106 RRID: SCR_008539) and cDNA prepared with the TaqMan Reverse Transcription System (Applied Biosystems #N8080234). SYBR Green-based real time quantitative PCR (RT-qPCR) was performed using primers for c-Myc, Bcl-2, NKLAM, Rae-1, NKG2D, granzyme B, perforin and 18S rRNA (as a control) purchased from Integrated DNA Technologies (IDT: PrimeTime qPCR primers RRID: SCR_025813) or Qiagen (RT^2^ qPCR primers). Samples were run on an ABI 7500 Real-Time PCR System (RRID: SCR_018051).

### Immunoblotting

HEK293 cells were purchased from ATCC (RRID: CVCL 0045). They were maintained in DMEM media (HyClone SH3024.02) with 10% fetal bovine serum (R&D S11150) and tested negative for mycoplasma contamination every 6 months. Cells were transfected with either 0.5 or 1.0 μg of plasmid containing NKLAM (pcDNA-CMV-NKLAM) using Lipofectamine 3000 (Invitrogen L3000008) and incubated overnight. Cell lysates were prepared as described, separated using SDS-PAGE and transferred to PVDF membranes (10). Blots were probed for NKLAM, myc and Bcl-2. β-actin served as the loading control. Blots were imaged using the Bio-Rad Chemidoc Gel Imaging System (RRID: SCR_019684) Densitometry analyses of immunoblots were performed with Image Lab v 6.0.1 software (Bio-Rad RRID: SCR_014210). Blots were probed using antibodies to β-actin (Sigma-Aldrich #A5441 RRID: AB_476744), c-Myc (Cell Signaling #5605S RRID: AB_1903938) and Bcl-2 (Cell Signaling #2876 RRID: AB_2064177). The anti-NKLAM antibody, produced in house, has been described previously (8, 9).

### Statistical analysis

*p* values were calculated using the Student’s *t* test, 2-way ANOVA or Chi-Square in Excel. Kaplan-Meier curves of tumor-free survival were generated in SPSS; *p* values were obtained with the log-rank Mantel-Cox test.

## Results

### Lymphoma development is accelerated in NKLAM KO Eμ-myc mice

**Figure 1:**
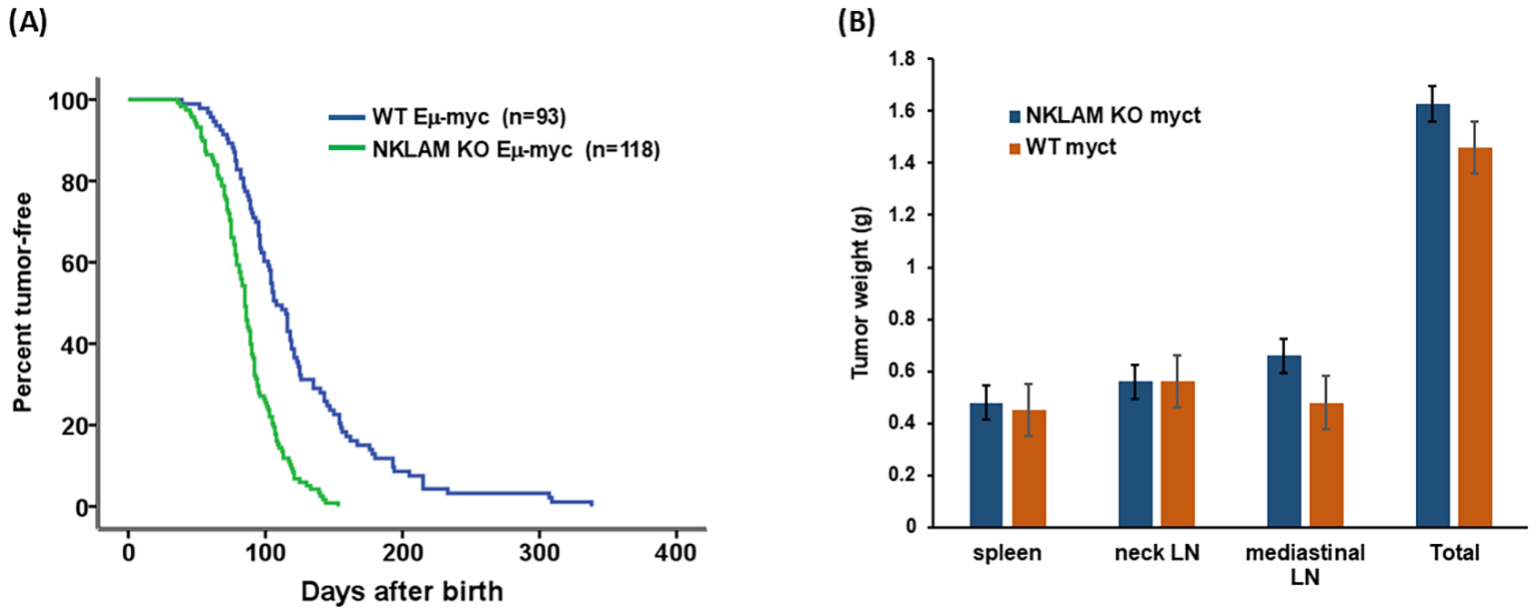
Lymphoma development is accelerated in NKLAM KO Eμ-myc mice. **(A)** NKLAM KO Eμ-myc mice (n=93) develop lymphoma faster (mean: 86 days) than WT Eμ-myc mice (n=118) (mean: 123 days) (*p* = 1.2x10^-5^). **(B)** Tumor weights in spleen, neck and mediastinal lymph nodes (LN) are comparable between NKLAM KO and WT Eμ-myc (myct) mice (n≥48 per group).

NKLAM KO and WT Eμ-myc mice were monitored for tumor growth, typically manifesting in enlarged lymph nodes. Tumor development was significantly accelerated in NKLAM KO Eμ-myc mice (mean 86 days) compared with WT Eμ-myc mice (mean 123 days) (**Figure 1A**). Upon sacrifice, spleens and tumorinvolved lymph nodes from the neck and mediastinum were weighed. There was no difference in the weights of tumor-bearing organs between NKLAM KO and WT Eμ-myc (myct) mice (**Figure 1B**).

### Pre-B cell expansion and persistence are greater in young NKLAM KO Eμ-myc mice

**Figure 2:**
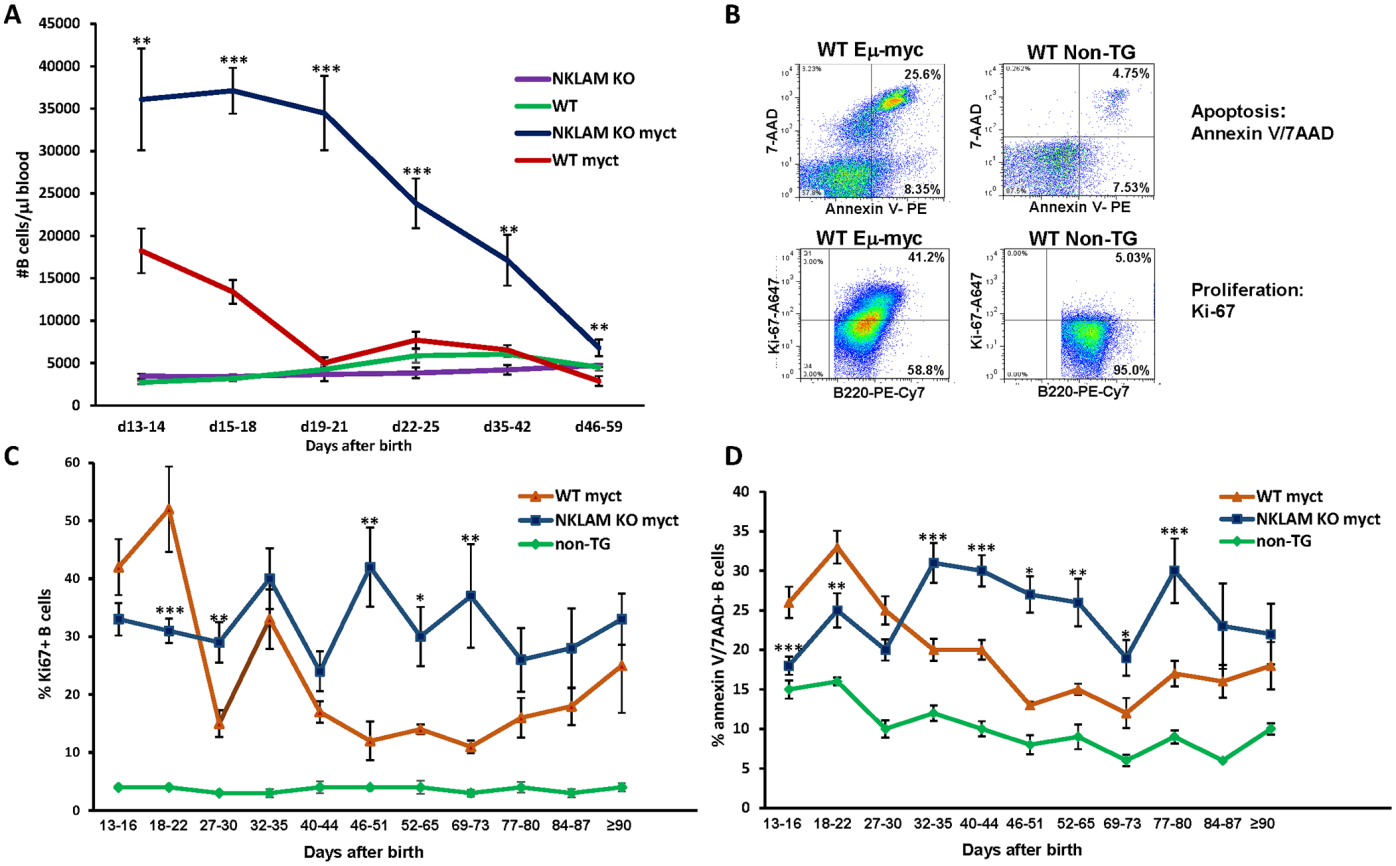
Early pre-B cell expansion and persistence are greater in young NKLAM KO Eμ-myc (myct) mice than in WT myct mice. **(A)** Peripheral blood from WT and NKLAM KO transgenic (myct) and non-transgenic mice was obtained between 13 and 59 days after birth. B cells within the CD45 population were identified by CD19 or B220 expression. Fluorescent Accucheck counting beads were added to cells for quantitation of total cell numbers (**p ≤ 0.01; ***p < 0.001, comparing NKLAM KO myct to WT myct mice at each age). (2-way ANOVA: *p* < 0.02) **(B)** Representative flow cytometry plots of annexin V/7AAD and Ki-67 expression in the pre-B cells in the blood of WT Eμ-myc and non-transgenic (TG) WT mice. **(C)** Percentage of Ki-67-positive B cells in the blood of myct mice between 13 and 94 days after birth (*p < 0.04; **p < 0.002; ***p < 0.001, comparing NKLAM KO myct to WT myct mice at each age) (2-way ANOVA: *p* < 0.05). **(D)** Percentage of annexin V ± 7-AAD+ pre-B cells in the blood of non-TG and myct mice between 13 and 94 days after birth (*p < 0.05; **p < 0.01; ***p ≤ 0.001, comparing NKLAM KO myct to WT myct mice at each age) (2-way ANOVA: *p* = 0.04). (n=9–26 for WT and NKLAM KO myct mice; n=35 for non-TG WT and NKLAM KO mice).

Lymphomagenesis begins with an early expansion of non-malignant B lineage cells in the bone marrow, blood, spleen and lymphoid organs; these cells disappear within a few weeks after birth due to massive apoptosis. To determine whether there was a difference between NKLAM KO and WT Eμ-myc mice during this phase, we quantitated B cells in the blood from mice 13–59 days after birth. B cells were identified as CD19+ CD45+ or B220+ CD45+; these are markers expressed on B cells as early as the pro-B cell stage and persist through the mature B cell stage. Absolute counts were calculated using fluorescent counting beads. Non-transgenic NKLAM KO and WT mice had comparable numbers of B cells. In contrast, NKLAM KO myct mice had significantly more pre-malignant B cells in the blood than their WT myct counterparts, and these cells persisted several weeks longer (**Figure 2A**).

The pre-B cell expansion is mediated by myc-induced proliferation, followed by apoptosis. We evaluated the proportions of proliferating pre-B cells by Ki-67 staining and apoptotic/dying pre-B cells in the blood of NKLAM KO and WT Eμ-myc by annexin V/7-AAD staining throughout the first 3 months of age. Representative flow plots of peripheral blood B cells in WT Eμ-myc transgenic and non-transgenic mice are shown in **Figure 2B**. In 13–22 day old mice, there were significantly more CD19+ Ki-67+ cells in WT Eμ-myc mice than in NKLAM KO Eμ-myc mice (**Figure 2C**). Over time, the percentage of CD19+ Ki-67+ cells in NKLAM KO Eμ-myc mice exceeded the number in WT Eμ-myc mice and remained higher over 90 days. Similarly, the proportion of CD19+ annexin V/7-AAD+ cells was higher in WT Eμ-myc mice than in NKLAM KO Eμ-myc mice at the youngest ages (13–30 days old) but NKLAM KO Eμ-myc mice maintained higher levels of apoptotic cells over time (**Figure 2D**). The combined result, shown in **Figure 2A**, was a larger number of pre-B cells in the blood of NKLAM KO Eμ-myc mice that remained elevated longer than in WT Eμ-myc mice.

**Figure 3:**
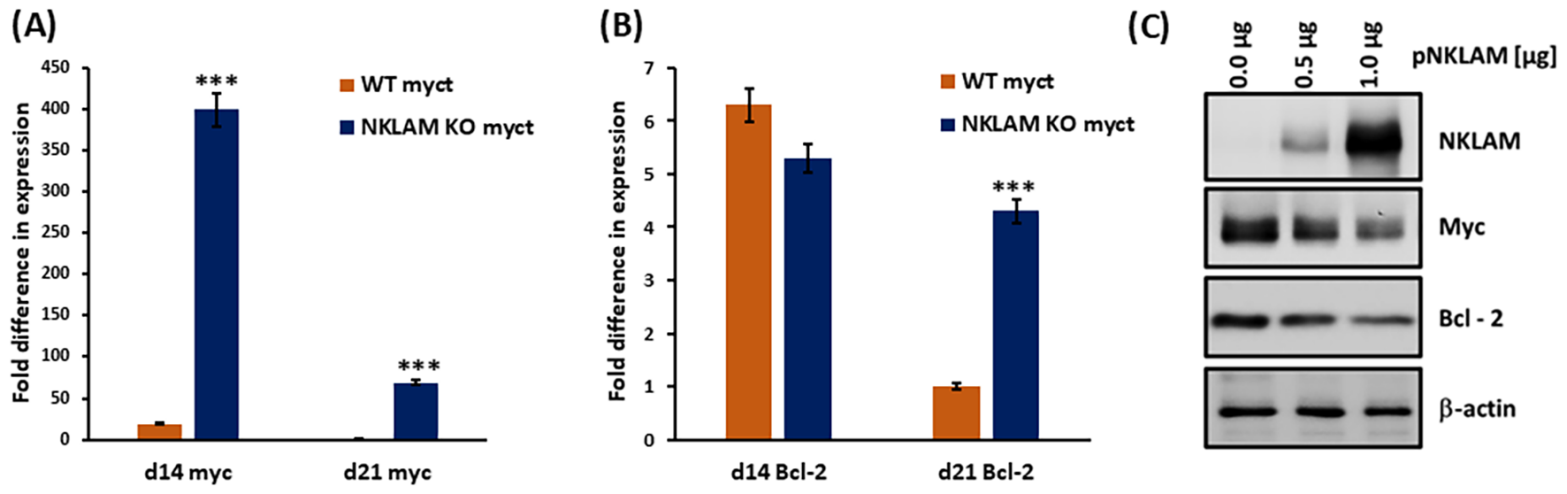
Pre-B cells from NKLAM KO myct mice have higher levels of c-myc and Bcl-2 than WT Eμ-myc (myct) mice. CD19+ peripheral blood cells from 14- and 21-day old NKLAM KO and WT myct mice were evaluated for levels of c-myc mRNA **(A)** and Bcl-2 mRNA **(B)** by RT qPCR. 18S rRNA was utilized as a housekeeping control (***p ≤ 0.001, comparing WT myct to NKLAM KO myct mice at each age) (n=9–13 per -group). **(C)** Representative immunoblot of myc and Bcl-2 protein levels in HEK293 cells transfected with NKLAM-expressing plasmid DNA. β-actin is the protein loading control.

Levels of myc mRNA in CD19+ B cells in the blood of NKLAM KO and WT Eμ-myc mice were evaluated. There was significantly more myc in the pre-B cells of 14- and 21-day old NKLAM KO Eμ-myc mice than in WT Eμ-myc mice (**Figure 3A**). This could account for their increased numbers and persistence. We examined Bc2 mRNA levels in these pre-malignant B cells since Bcl-2 has anti-apoptotic activity. On day 14, there was no difference in Bcl-2 levels between NKLAM KO and WT Eμ-myc B cells. By day 21, Bcl-2 was significantly higher in NKLAM KO Eμ-myc mice. This would account for their lower levels of apoptotic cells through day 22. (**Figure 3B**).

These findings suggest that NKLAM plays a direct role in controlling the levels of c-myc and Bcl-2 in these cells. We detected low levels of NKLAM mRNA in day 13 and day 21 pre-B cells in WT Eμ-myc mice; 4–8 times less than the amount found in the CD19- lymphocyte compartment. NKLAM protein was weakly expressed in these non-malignant B cells (**Supplementary Figure 1**). To further explore the role of NKLAM in this pre-B cell expansion, we examined myc and Bcl-2 levels in HEK293 cells transfected with different concentrations of plasmid DNA encoding NKLAM. There was a dose-dependent decrease in the endogenous levels of myc and Bcl-2 protein with increasing amounts of NKLAM (**Figure 3C**). These studies suggest that NKLAM regulates levels of myc and Bcl-2 in the non-malignant early B cells in myc transgenic mice. This results in a greater number of pre-B cells and longer persistence.

**Figure 4:**
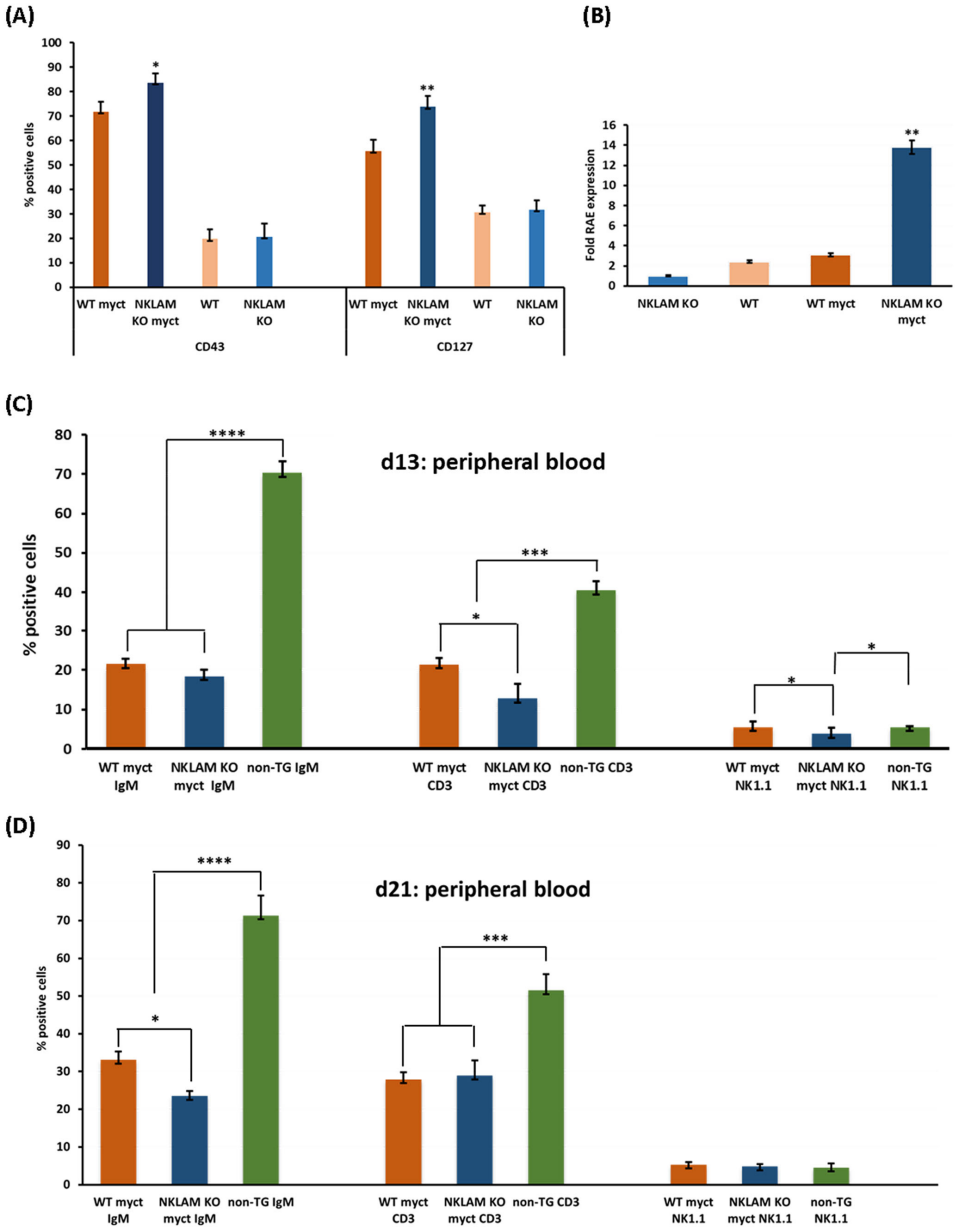
Peripheral blood from young NKLAM KO myct mice have more CD43+, CD127+ pre-B cells, more RAE-1 expression and fewer immunoregulatory cells than WT myct mice. **(A)** Percentage of CD43+ and CD127+ cells within the CD19+ B cell population was evaluated by flow cytometry (*p < 0.05; **p = 0.01, comparing WT myct to NKLAM KO myct) (n=9–14 per group). **(B)** Levels of RAE mRNA in CD19+ pre-B cells were assessed by qPCR (**p = 0.01, comparing WT myct to NKLAM KO myct) (n=8–11 per group). Fewer immunoregulatory cells are in the peripheral blood of young NKLAM KO myct mice. CD45+ blood cells from **(C)** 13-day and **(D)** 21-day old WT myct, NKLAM KO myct and non-transgenic (non-TG) mice were stained for expression of IgM, CD3 and NK1.1. (n=6–15 per group) (*p ≤ 0.04; ***p < 0.001; ****p < 0.0001).

We compared the phenotype of pre-malignant B cells in NKLAM KO and WT Eμ-myc mice. This polyclonal population is heterogeneous in its state of differentiation (25, 26). CD43 (leukosialin) is expressed by pro-B cells and is lost upon differentiation to pre-B and immature B cells. CD127 (IL-7Rα) is expressed at the earliest B lymphocyte progenitor stage and continues throughout the pre-B cell stage (26). NKLAM KO Eμ-myc mice had a higher percentage of CD43+ and CD127+ B cells 15 days after birth than WT Eμ-myc mice (**Figure 4A**). This suggests that pre-malignant B cells in young NKLAM KO myct mice are less differentiated than those in WT myct mice.

Rae-1 is induced during myc-driven B cell lymphomagenesis and is the dominant ligand of the NK receptor NKG2D on Eμ-myc lymphomas (27). We quantitated RAE-1 mRNA in CD19+ B cells from 14-day old NKLAM KO and WT non-transgenic and transgenic mice. RAE-1 levels were very low in non-transgenic and WT Eμ-myc B cells. There was 4-5-fold more RAE-1 mRNA in NKLAM KO Eμ-myc pre-malignant B cells than in WT transgenic B cells (**Figure 4B**).

The percentage of CD19+ B cells expressing surface μ heavy chain (IgM+) was the same in both NKLAM KO and WT Eμ-myc mice on day 13, and significantly lower than in non-transgenic mice (**Figure 4C**). The percentage of CD3+ T cells and CD3- NK1.1+ NK cells was also lower in the blood of NKLAM KO Eμ-myc mice than in WT myct+ mice, suggesting impaired immunity. There were fewer T cells overall in Eμ-myc mice compared to non-transgenic mice. By day 21, the percentage of CD19+ IgM+ B cells remained lower in NKLAM KO than in WT Eμ-myc mice; both remained significantly lower than in non-transgenic mice. T cell levels were similar between NKLAM KO and WT myct+ mice but were lower than that seen in non-transgenic mice. By day 21, NK populations were equivalent in all groups (**Figure 4D**).

### Lymphomas that arise in NKLAM KO Eμ-myc mice are more differentiated than lymphomas in WT Eμ-myc mice

**Figure 5:**
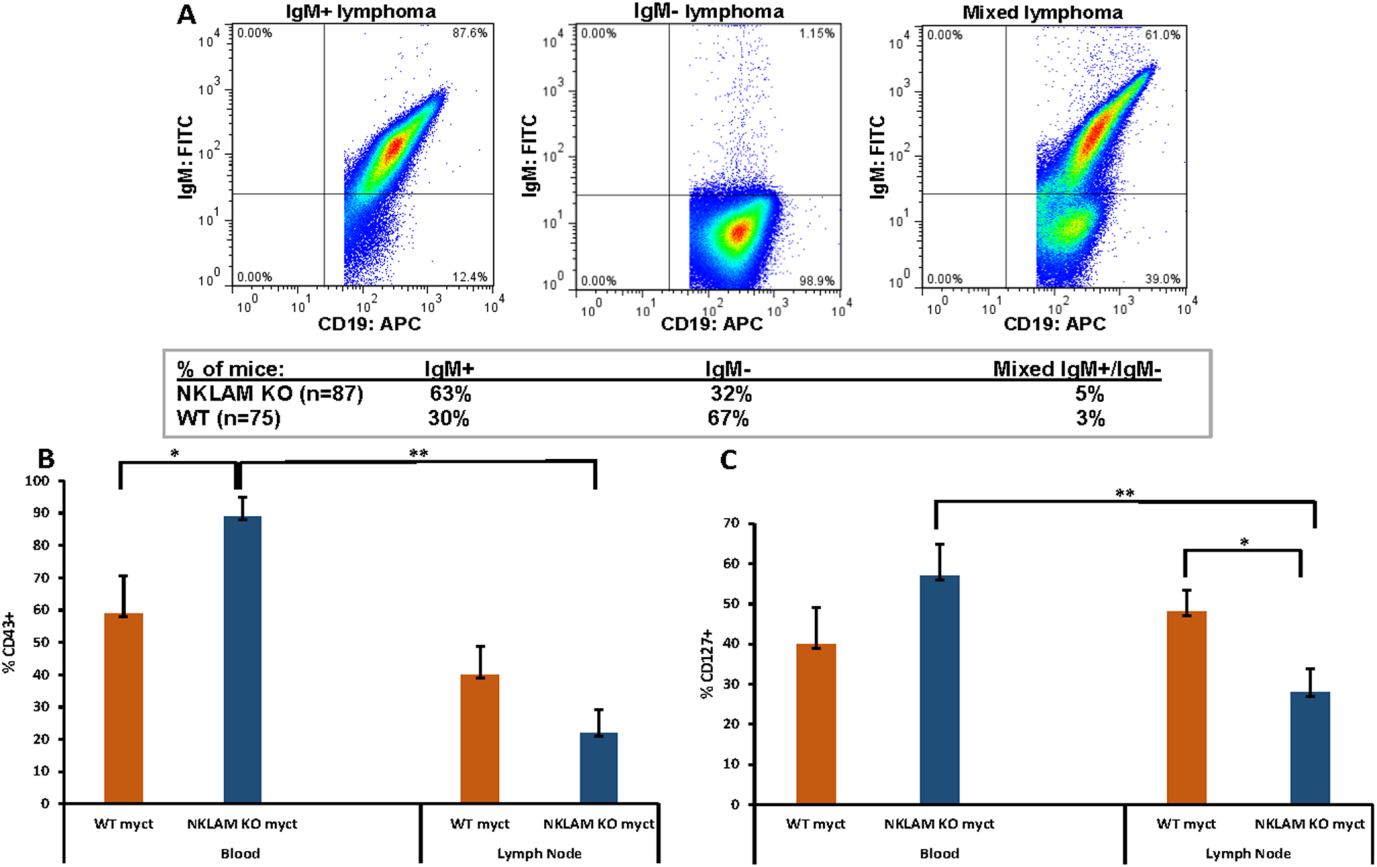
B cell lymphomas arising in NKLAM KO Eμ-myc mice are more differentiated than lymphomas in WT Eμ-myc mice. **(A)** Flow cytometry plots of representative IgM+, IgM- and mixed IgM+/IgM- lymphomas. CD19 is on the x-axis and IgM is on the y-axis. Shown is the percentage of NKLAM KO myct and WT myct mice with IgM+, IgM- and mixed IgM+/IgM- lymphomas. **(B)** Percentage of CD19+/CD43+ cells and **(C)** CD19+/CD127+ cells in the blood and lymph nodes of tumor-bearing WT myct and NKLAM KO myct mice (n=11–13 per group) (*p < 0.05; **p ≤ 0.01).

Myc drives early pre-B cell expansion in lymphoid organs; progression to lymphoma requires a second genetic alteration, often loss of p53 or dysregulation of Bcl-2 (28). Lymphomas in Eμ-myc mice may arise from pre-B, pro-B or immature B cells. We evaluated the phenotype of lymphomas from NKLAM KO and WT Eμ-myc mice. Representative flow plots and percentages of mice with each phenotype are shown in **Figure 5A**. The majority (67%) of B cell lymphomas in WT Eμ-myc mice were surface μ heavy chain negative (denoted IgM-) pre-B cells, consistent with published findings (25, 29). In contrast, lymphomas in NKLAM KO Eμ-myc mice were more mature, predominantly surface μ heavy chain positive (63% were IgM+). These findings suggest that NKLAM influences the differentiation of lymphomas in Eμ-myc mice.

### Phenotypic differences in B cell lymphomas from NKLAM KO and WT Eμ-myc mice

NKLAM KO and WT Eμ-myc tumor cells were examined for expression of CD43 and CD127. As shown in **Figure 5B**, >85% of the CD19+ B cells in the blood of tumor-bearing NKLAM KO Eμ-myc mice were CD43+, significantly higher than that seen in WT Eμ-myc blood. The reverse was seen in lymph node tumor cells, with a lower percentage of positive cells overall, and lowest in NKLAM KO Eμ-myc mice. A similar pattern was seen with CD127 (**Figure 5C**). This suggests that lymph node tumor cells are more mature, while those in the blood are more immature. The most mature B cells were found in tumor-bearing lymph nodes of NKLAM KO Eμ-myc mice, consistent with their more differentiated tumor phenotype.

Expression of MHC class I and NKG2D ligands (NKG2DL) was highly heterogeneous and not significantly different between NKLAM KO and WT Eμ-myc tumors. However, when stratified into IgM+ and IgM- phenotypes, there were significantly higher levels of NKG2DL on IgM+ NKLAM KO tumors compared with all other groups. Surface expression of NKG2DL was 40% higher on NKLAM KO IgM+ Eμ-myc tumors than on any other group (*p* < 0.05). A similar observation was made with MHC class I, with IgM+ NKLAM KO tumors having the highest levels, although not to the level of significance.

**Figure 6:**
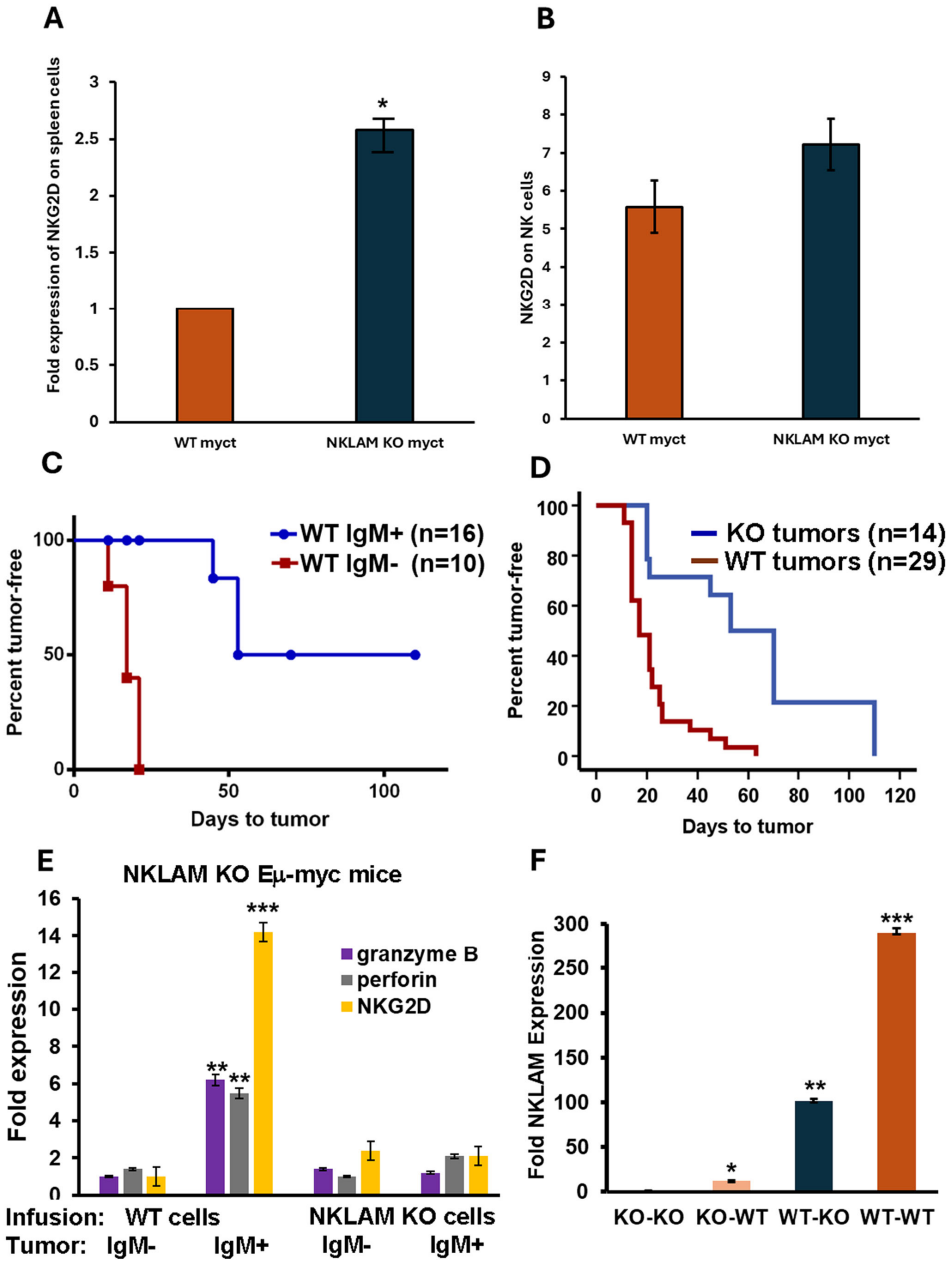
Immune cells modulate lymphoma immunogenicity and phenotype. **(A, B)** Splenic lymphocytes from tumor-bearing NKLAM KO myct mice express more NKG2D than WT myct mice. **(A)** Expression of NKG2D by qPCR analysis (WT myct n=21; NKLAM KO myct n=33) (*p = 0.03). **(B)** Flow cytometry analysis of NKG2D expression on CD3-/NK1.1+ splenic lymphocytes from tumor-bearing mice. Results are presented as the fold difference in NKG2D levels compared to the corresponding non-NK cell population (WT myct n=29; NKLAM KO myct n=42) (*p* = 0.1). **(C, D)** IgM- WT lymphomas are more tumorigenic than IgM+ tumors. **(C)** WT non-transgenic mice were injected with either WT IgM- or WT IgM+ Eμ-myc tumor cells and monitored for tumor development (*p* = 5 x10^-10^). **(D)** WT non-transgenic mice were injected with either WT Eμ-myc lymphoma cells (WT) or NKLAM KO Eμ-myc (NKLAM KO) tumor cells and monitored for tumor development. (*p* < 0.00002). **(E, F)** Higher levels of immune gene expression in IgM+ tumors after infusion of WT splenic lymphocytes into NKLAM KO Eμ-myc mice. **(E)** Tumors from WT or NKLAM KO- non-transgenic lymphocyte-injected NKLAM KO myct mice were analyzed for transcripts of cytolytic proteins granzyme B and perforin and NK receptor NKG2D by qPCR. Tumors were grouped by IgM status (n=7–15 per group) (**p < 0.01; ***p < 0.0001, comparing all groups). **(F)** NKLAM mRNA levels in tumors from Eμ-myc mice injected with non-transgenic lymphocytes. KO-KO: tumors from NKLAM KO myct mice injected with non-transgenic NKLAM KO lymphocytes (n=3); KO-WT: tumors from WT Eμ-myc mice injected with non-transgenic NKLAM KO lymphocytes (n=5). WT-KO: tumors from NKLAM KO Eμ-myc mice injected with non-transgenic WT lymphocytes (n=8); WT-WT: tumors from WT Eμ-myc mice injected with non-transgenic WT lymphocytes (n=4) (*p < 0.001; **p < 0.0005; ***p < 0.0001).

Due to the differences in NKG2DL levels between IgM+ and IgM- NKLAM KO tumors, we evaluated splenic lymphocytes from tumor-bearing mice for expression of the NKG2DL receptor, NKG2D. Lymphocytes from NKLAM KO Eμ-myc mice expressed significantly higher levels of NKG2D mRNA than WT transgenic mice (**Figure 6A**). Flow cytometry analysis indicated more NKG2D expression on NKLAM KO Eμ-myc CD3- NK1.1+ NK cells than on WT Eμ-myc NK cells, but did not reach the level of significance (*p* = 0.1) (**Figure 6B**).

### IgM- WT lymphomas are more tumorigenic than IgM+ tumors

A subset of WT Eμ-myc mice succumbed to disease early, after less than 80 days. Greater than 90% of them had IgM- lymphomas, compared with 67% overall (**Table 1**). NKLAM KO Eμ-myc mice with tumor-free survival less than 80 days had the same distribution of IgM- and IgM+ lymphomas as the entire group. This suggests that IgM- lymphomas in WT Eμ-myc mice are more aggressive than IgM+ lymphomas.

**Table 1:** Distribution of IgM- and IgM+ lymphomas in NKLAM KO and WT myct mice.

Survival (days)	NKLAM KO myct		WT myct	
	% IgM+	% IgM	% IgM+	% IgM-
≤ 80	59	41	10	90*
n	13	9	10	10
Overall survival	63	32	30**	67
n	55	28	23	59

*p = 0.009, comparing the number of WT and NKLAM KO IgM- lymphomas.

**p < 0.0001, comparing the number of WT and NKLAM KO IgM+ lymphomas.

To test this, non-transgenic WT mice were injected with either WT Eμ-myc IgM- or IgM+ tumor cells. As shown in **Figure 6C**, mice receiving WT IgM- tumor cells succumbed to disease much faster than those injected with WT IgM+ tumor cells. Tumor growth in WT non-transgenic mice injected with WT Eμ-myc lymphomas was also significantly accelerated compared to growth of NKLAM KO Eμ-myc tumor cells (**Figure 6D**). Collectively, these results indicate that NKLAM influences not only the final differentiation state of lymphomas but also their tumorigenicity. This may, in part, reflect a defect in immunoediting in NKLAM KO Eμ-myc mice, due to reduced NK and/or T cell activity.

### Infusions of WT splenic lymphocytes into NKLAM KO Eμ-myc mice influence tumor phenotype

To determine whether infusion of WT immune cells would slow the progression of disease in NKLAM KO Eμ-myc mice, splenic lymphocytes (4–5 x 10^7^ cells) from non-transgenic WT or NKLAM KO mice were injected iv into NKLAM KO Eμ-myc mice every other week, starting 4 weeks after birth. Adoptive transfer of either WT or NKLAM KO lymphocytes had no effect on tumor-free survival (**Supplementary Figure 2**). However, there was a dramatic effect on the phenotype of tumors that developed when WT lymphocytes were administered. In the majority (67%) of NKLAM KO Eμ-myc mice injected with WT cells, the tumor phenotype shifted from surface IgM+ to IgM-, which is the predominant phenotype seen in WT Eμ-myc mice. This did not occur in mice receiving NKLAM KO spleen cells (**Table 2**). This suggests that NKLAM+ WT lymphocytes influence/inhibit tumor differentiation.

**Table 2:** Adoptive transfer of WT or NKLAM KO lymphocytes into NKLAM KO myct mice.

Tumor distribution	% IgM+	% IgM-	n
NKLAM KO myct mice			
No cells transferred	63	32	87
Transfer NKLAM KO cells	64	36	26
Transfer WT cells	38	62**	33
WT myct mice			
No cells transferred	30	67	75

**p < 0.005, comparing transfer of NKLAM KO and WT cells into NKLAM KO myct mice.

We speculated that interaction between NKLAM KO lymphoma cells and infused WT lymphocytes was responsible for this shift in phenotype. To test this, tumors from WT lymphocyte-injected KO mice were evaluated for infiltrating cytotoxic cells by measuring transcripts of NKG2D and cytolytic proteins granzyme B and perforin by qPCR. IgM- tumors from WT cell-injected mice and all tumors from NKLAM KO lymphocyte-injected mice expressed similar amounts of all transcripts (**Figure 6E**). However, NKG2D mRNA was significantly elevated (14-fold) in IgM+ tumors from WT lymphocyte-injected mice. IgM+ tumors from WT spleen cell-injected NKLAM KO Eμ-myc mice also had 6- and 5-fold higher levels of granzyme B and perforin mRNA, respectively.

Tumors were examined for NKLAM by qPCR (**Figure 6F**). When NKLAM KO spleen cells were injected into WT Eμ-myc mice (KO-WT), tumors had detectable levels of NKLAM, suggesting infiltration of host immune cells. NKLAM expression was even greater in tumors from NKLAM KO Eμ-myc mice injected with WT spleen cells (WT-KO), indicating that infused spleen cells and/or their progeny infiltrated the tumors. Highest NKLAM levels were seen in tumors from WT Eμ-myc mice injected with WT spleen cells (WT-WT). These results are consistent with a role for NKLAM+ immune cells in shaping lymphoma development via immune regulation.

**Figure 7:**
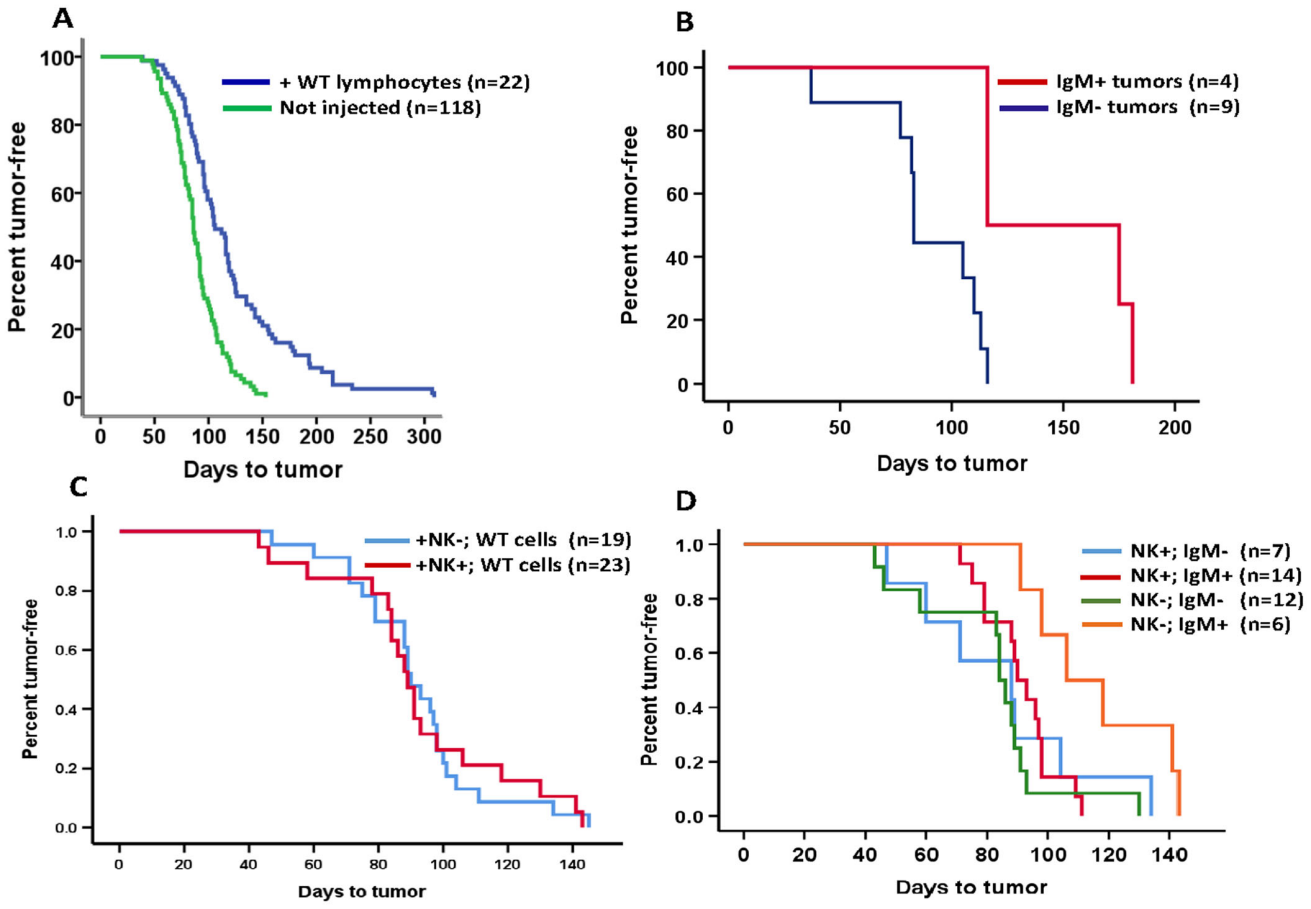
Infusion of WT lymphocytes into NKLAM KO Eμ-myc mice influences time to tumor development and tumor phenotype. **(A, B)** Injection of WT splenic lymphocytes into the livers of 10–11-day old NKLAM KO Eμ-myc mice, followed by one iv injection of cells 2 weeks later, delays tumor development. **(A)** Tumor development in WT lymphocyte-injected NKLAM KO myct mice (blue) and in non-injected NKLAM KO myct mice (green) (*p* < 0.01). **(B)** Tumor development is slower in WT lymphocyte injected NKLAM KO Eμ-myc mice that develop IgM+ tumors (red) compared to those that develop IgM- tumors (blue) (*p* = 0.006). **(C, D)** Effect of adoptive transfer of DX5+ NK cells (CD3- NK1.1 population) or the lymphocytes remaining after NK positive selection from spleens of non-transgenic WT mice into NKLAM KO Eμ-myc mice, starting 13 days after birth, on tumor-free survival. **(C)** Time to tumor development is similar in WT NK+ (red) and WT NK- (blue) injected NKLAM KO Eμ-myc mice. **(D)** NKLAM Eμ-myc mice that develop IgM+ lymphomas (orange) after injection with NK- WT spleen cells are tumor-free longer than all other groups. NK+, IgM- tumors (blue); NK+, IgM+ tumors (red); NK-, IgM- tumors (green); NK-, IgM+ tumors (orange) (*p* = 0.02, comparing NK-, IgM+ to all other groups).

We injected WT lymphocytes into the livers of 10–11-day old NKLAM KO Eμ-myc mice, with an additional injection 2 weeks later, to determine whether earlier administration of WT cells would enhance survival of NKLAM KO Eμ-myc mice. Mean tumor-free survival increased to 103 days, from 86 (**Figure 7A**). A shift in tumor phenotype was also observed; 64% of these tumors were IgM-, twice the number of the non-injected group. Mean survival of mice with IgM+ tumors was 147 days, significantly longer than IgM- tumor-bearing mice (mean 90 days) (**Figure 7B**). These results indicate that infusion of NKLAM+ WT lymphocytes into NKLAM KO Eμ-myc mice soon after birth modulates tumor phenotype and tumorigenicity.

### Infusions of WT NK cells into NKLAM KO Eμ-myc mice influence tumor phenotype

To determine which WT lymphocyte subsets were responsible for increasing the tumor-free survival of NKLAM KO myc mice and altering their tumor phenotype, WT lymphocytes were separated into DX5+ NK cells (CD3- NK1.1 population), or lymphocytes remaining after NK positive selection. These were injected into NKLAM KO Eμ-myc mice every other week, starting 13 days after birth. There was no difference in tumor-free survival between the 2 groups, and no increase in survival compared to untreated NKLAM KO Eμ-myc mice (**Figure 7C**). However, a significantly higher percentage of WT NK+ -infused mice developed IgM+ lymphomas (75% versus 63%). Conversely, injections of non-NK WT lymphocytes significantly shifted the tumor phenotype towards IgM- (**Table 3**). These results suggest that NKLAM+ NK cells promote differentiation of tumor cells while NKLAM+ non-NK cells promote IgM- lymphoma development.

**Table 3:** Adoptive transfer of WT NK+ or NK- lymphocytes into NKLAM KO myct mice.

Tumor distribution	% IgM+	% IgM-	n
NKLAM KO myct			
No cells transferred	63	32	87
Transfer NK+ WT cells	75	25*	23
Transfer NK- WT cells	33	67**	19
WT myct			
No cells transferred	30	67	75

*p = 0.006, comparing transfer of NK- and NK+ WT cells into NKLAM KO myct mice.

**p = 0.003, comparing transfer of NK- cells with no cells transferred into NKLAM KO myct mice.

Tumor-free survival was highest in WT NK+ or NK- lymphocyte injected NKLAM KO Eμ-myc mice that developed IgM+ tumors (mean survival 99 days versus 82 days for mice with IgM- tumors). Mice with the longest tumor-free survival were non-NK lymphocyte-injected NKLAM KO Eμ-myc mice with IgM+ tumors (mean survival 116 days) (**Figure 7D**). There were no differences in expression of NKG2DL or MHC class I among tumor groups, although there were wide variations in the levels. Therefore, it remains to be determined what additional factors contribute to tumorigenicity and immunogenicity.

## Discussion

**Figure 8:**
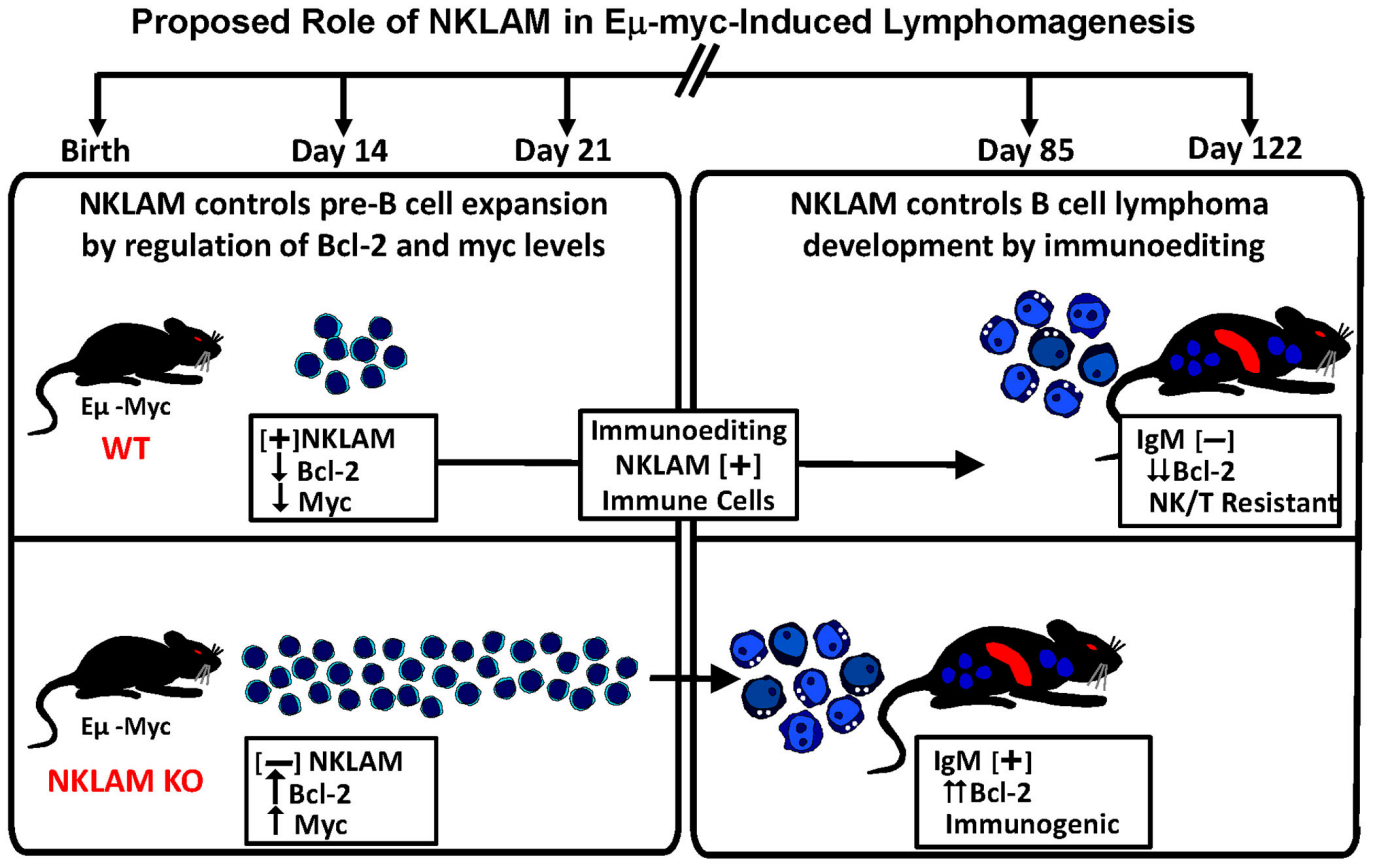
NKLAM influences both the early and late phases of myc-driven B cell lymphomagenesis. During the pre-malignant B cell expansion phase, the presence of NKLAM slows their proliferation and/or limits the persistence of these cells before they disappear from the periphery. During the second lymphomagenesis phase, NKLAM-expressing immune cells modulate the immunophenotype of lymphomas that arise by immunoediting, resulting in emergence of immune-resistant variants.

These studies uncover a role for NKLAM in tumorigenesis. Previous experiments revealed that tumor dissemination and metastasis are accelerated in NKLAM KO mice (21). Here we show that NKLAM influences both the early and late phases of myc-driven B cell lymphomagenesis. During the pre-malignant B cell expansion phase, the presence of NKLAM slows their proliferation and/or limits the persistence of these cells before they disappear from the periphery. During the second lymphomagenesis phase, our results suggest that NKLAM-expressing immune cells modulate the immunophenotype of lymphomas that arise, resulting in emergence of immune-resistant variants. This model is depicted in **Figure 8**.

Levels of c-myc and Bcl-2 are higher in the non-malignant B lineage cells of young NKLAM KO Eμ-myc mice than in young WT Eμ-myc mice, which would account for their increased numbers and persistence. Consistent with this is the finding that there is a dose-dependent decrease in c-myc and Bcl-2 protein levels in HEK293 cells with increased NKLAM expression. We have shown previously that NKLAM reduces the half-life of c-myc protein (13), which would contribute to the lower levels of pre-B cells in WT Eμ-myc mice compared to NKLAM KO myct mice during the first few months after birth. Many RBR E3 ubiquitin ligases affect proteins associated with apoptosis and mitophagy. RBR E3 ligase IBRDC2 regulates Bax expression; Parkin mono-ubiquitinates Bcl-2 and interacts with the mitochondrial kinase PINK1 (19, 30, 31). Studies are in progress to investigate how NKLAM affects both transcription and translation of myc and Bcl-2, whether by direct ubiquitination and/or indirectly by influencing expression of other participants in these pathways of proliferation and apoptosis.

Tumor development is accelerated in NKLAM KO Eμ-myc mice compared to WT Eμ-myc mice. We attribute this to the persistence of pre-malignant B cells as well as diminished immune function. Most B cell lymphomas in WT Eμ-myc mice are surface IgM- pre-B cells; the lymphomas in NKLAM KO Eμ-myc mice are predominantly surface IgM+ B cells. This suggests that NKLAM influences the differentiation state of lymphomas.

A similar phenotype is seen in Eμ-myc mice lacking the BH3-only proapoptotic Bcl-2 family member Bcl-2 modifying factor (Bmf). Bmf deficiency results in a 10-fold increase in the number of pre-malignant B cells in young mice, increases the proportion of IgM+ tumors from 30% to 67%, and accelerates tumorigenesis (32). Absence of the p53 homolog p73 in Eμ-myc mice increases the proportion of mice that develop IgM+ B cell lymphomas but does not affect survival (33). In contrast, loss of a single allele of the gene encoding the E6-associated protein E3 ubiquitin ligase significantly extends the survival of Eμ-myc mice, lowers the numbers of circulating pre-malignant B cells due to induction of cellular senescence, but increases the proportion of IgM+ tumors from 33% to 71% (25). A survey published in 2023 identified 48 genes with a critical impact on myc-induced lymphomagenesis; most are associated with apoptosis/proliferation, metabolism, epigenetic regulation and transcription/translation. NKLAM is one of a few immunoregulatory genes shown to control myc-driven lymphomagenesis (23).

Infusions of WT lymphocytes into NKLAM KO Eμ-myc mice shift the phenotype of tumor cells to IgM-, which is the predominant one seen in WT Eμ-myc mice. During the second hit phase, when B cells become tumorigenic, NKLAM-expressing lymphocytes (NK cells, T cells or both) modulate the phenotype and susceptibility to lysis of lymphomas that arise. Immunoediting by both innate and adaptive immune cells shapes the immunogenicity of tumors, promoting the development of less immunogenic clones that escape detection by the immune system (34–37). In Eμ-myc mice, the IgM- tumors that arise are more aggressive; mice with these tumors die early. They are also more aggressive than IgM+ tumors when injected into healthy mice.

IgM- tumors grow more quickly in non-transgenic WT mice and are less susceptible to NK-mediated killing than IgM+ lymphomas that arise in NKLAM KO Eμ-myc mice (**Figure 7**). These tumors are less immunogenic, expressing lower levels of the NKG2D ligand Rae-1 (**Figure 4B**). Schuster et al. found that high Bcl-2 levels in Eμ-myc-derived lymphomas are associated with increased susceptibility to cell-mediated cytolysis, which is consistent with our findings (38).

Infusions of WT non-transgenic spleen cells into NKLAM KO myct mice extend their survival (**Figure 7A**). We propose that this allows for immunoediting by NKLAM+ WT immune cells, which increases the proportion of mice that develop less immunogenic IgM- B cell lymphomas (**Table 2**). Neither purified NK cells nor NK-depleted lymphocytes are individually able to extend the survival of NKLAM KO Eμ-myc mice (**Figure 7C**). This suggests that both NK cells and T cells contribute to immunoediting of myc-driven B cell lymphomas. Consistent with this observation is the study by Croxford et al. demonstrating that the loss of early-stage B cells in young Eμ-myc mice depends on all three populations: CD4+ T cells, CD8+ T cells and NK cells (39).

The role of NKLAM in modulating lymphoma development is further supported by the higher levels of granzyme B, perforin, NKG2D and NKLAM in IgM+ lymphomas from WT myct mice (**Figure 6E**). These reflect the greater infiltration of NKLAM+ cytotoxic immune cells into these tumors and correlate with longer tumor-free survival of these mice.

Collectively, these studies uncover a role for NKLAM in both the early and late phases of lymphomagenesis in Eμ-myc mice. Ongoing investigations of NKLAM function may lead to the development of new strategies for both cancer prevention and treatment.

## Data Availability

The raw data supporting the conclusions of this article will be made available by the authors, without undue reservation.
