# Supplementary material for: E3 ubiquitin ligase NKLAM/RNF19b suppresses myc-driven B cell lymphomagenesis in Eμ-myc mice
Source: Front Oncol. 2026 Jul 15;16:1874366. doi: 10.3389/fonc.2026.1874366 (PMC13414219; doi:10.3389/fonc.2026.1874366)
Supplement: Supplementary file 1 [file DataSheet1.docx]

**Supplementary Figures and Legends**

**Figure S1**. Immunoblot of lysates of CD19+ pre-B cells from day 19 NKLAM KO and WT myc transgenic mice. The band just above 100 kD is NKLAM. The positive control is lysate from NK cells.

**Figure S2.** Time to tumor development is the same in NKLAM KO myct mice injected iv every other week with either PBS, 4-5 x 10^7^ WT or NKLAM KO splenic lymphocytes from non-transgenic mice starting 4 weeks after birth.

**Figure S1**

**
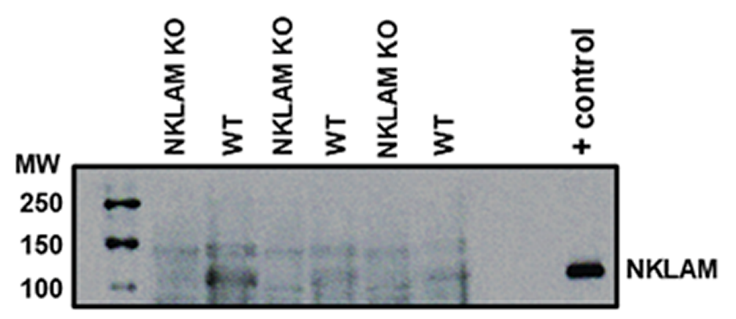
**

**Figure S2**

**
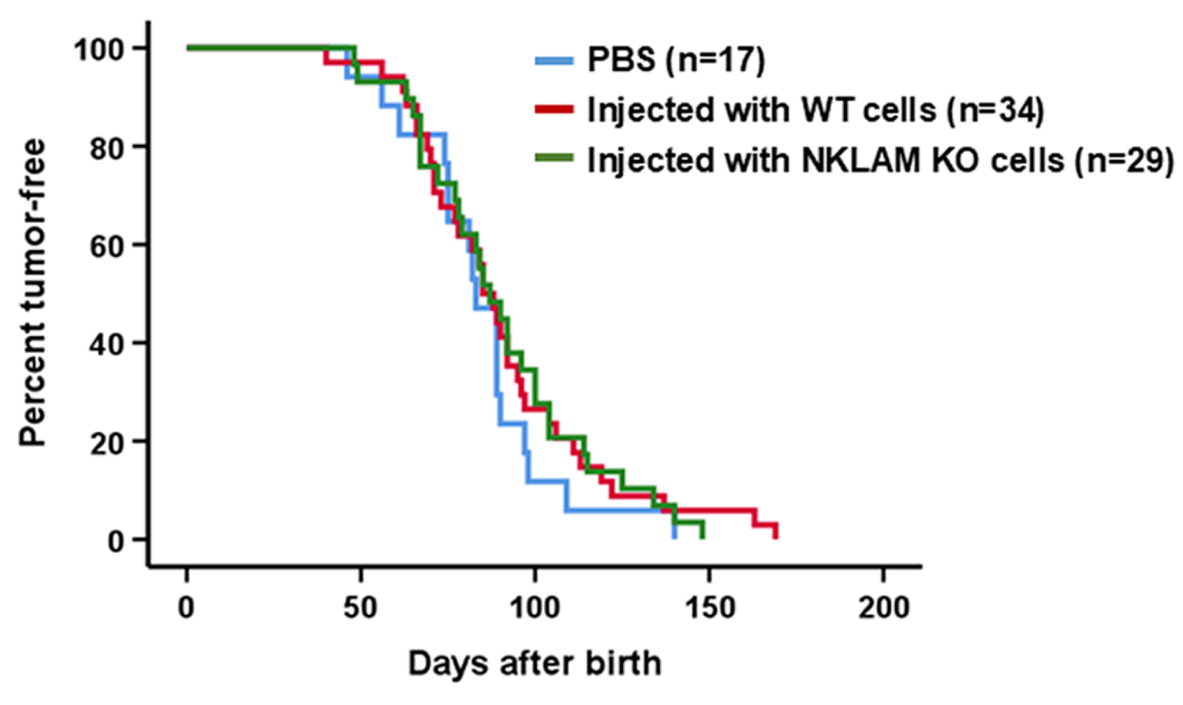
**
